# Do adolescents consider mind-body skills groups an acceptable treatment for depression: results from a pilot study

**DOI:** 10.1186/s12887-021-02942-3

**Published:** 2021-10-27

**Authors:** Lindsey D. Cunningham, Eduardo F. Salgado, Matthew C. Aalsma, Jennifer M. Garabrant, Julie K. Staples, James S. Gordon, Michelle P. Salyers

**Affiliations:** 1 Department of Psychology, Florida International University, 11200 SW 8th St, AHC5, Miami, FL 33199 USA; 2 Department of Psychology, Indiana University-Purdue University Indianapolis, 402 N. Blackford Street, LD 120B, Indianapolis, IN 46202 USA; 3 Department of Pediatrics – Adolescent Behavioral Health Research Program, Indiana University School of Medicine, 410 W. 10th Street, Suite 2025, Indianapolis, IN 46202 USA; 4 The Center for Mind-Body Medicine, 5225 Connecticut Avenue NW, Suite 414, Washington, DC 20015 USA

**Keywords:** Adolescent, Mind-body therapies, Patient acceptance of health care, Depressive disorder, Primary care setting

## Abstract

**Background:**

Mind-Body Skills Groups (MBSGs) have shown promise in reducing adolescent depression symptoms; however, little is known about adolescents’ perspectives on this treatment. The objective of this study was to understand the acceptability of a new treatment for depressed adolescents in primary care settings.

**Methods:**

Adolescents participating in a 10-week MBSG treatment were interviewed to understand their perspectives on the acceptability and effectiveness of the treatment. Interviews were collected at post-intervention and at a 3-month follow-up visit.

**Results:**

A total of 39 adolescents completed both the post-intervention and 3-month follow-up interview. At post-intervention and follow-up, 84% of adolescents stated the MBSGs helped them. When asked how the MBSGs helped them, 3 areas were identified: learning new MBSG activities and skills, social connection with others within the group, and outcomes related to the group. Many adolescents reported no concerns with the MBSGs (49% at post- intervention; 62% at follow-up). Those with concerns identified certain activities as not being useful, wanting the group to be longer, and the time of group (after school) being inconvenient. Most adolescents reported that their life had changed because of the group (72% at post-intervention; 61% at follow-up), and when asked how, common responses included feeling less isolated and more hopeful.

**Conclusions:**

Adolescents found the MBSGs to be helpful and acceptable as a treatment option for depression in primary care. Given the strong emphasis on treatment preference autonomy and the social activities within the group, MBSGs appear well-suited for this age group.

**Trial registration:**

NCT03363750; December 6th, 2017.

**Supplementary Information:**

The online version contains supplementary material available at 10.1186/s12887-021-02942-3.

## Background

Developing evidence-based treatments to reduce mental illness is key for improving and advancing the healthcare system [1]. However, before treatments can be properly implemented and practiced, they must first undergo numerous rounds of rigorous testing. The first step often begins with a pilot study, which explores the preliminary effectiveness, feasibility, and acceptability of treatments [2]. Acceptability in particular, is a key factor when designing and evaluating the success of treatment implementation, and is a strong predictor of patient satisfaction and trust, adherence, and health outcomes [3–5]. The term acceptability has been described many different ways but can be best defined as a multifaceted construct that assesses whether providers and consumers of an intervention have a positive response – both cognitively and emotionally - to a treatment [6]. Acceptability provides insight as to the appropriateness of a new treatment for a target population and predicts treatment utilization in clinical practice [7–9].

Adolescent depression is one area that warrants special attention for novel interventions given the substantial increase in rates of depression and suicide in the past decade [10–12]. Given biological and social changes that occur throughout puberty, risk of depression greatly increases [13]. Adolescents undergo physical, cognitive, and emotional changes throughout puberty that often provoke a desire to uncover their identity, establish autonomy, and build upon pre-existing notions of the world and the social frameworks within it [14]. Due to this rapid influx of hormonal changes and shift in awareness, youth are highly susceptible to developing symptoms of depression [15]. Recent findings suggest that the prevalence of adolescent depression is significant [16] but treatment adherence remains low [17–19]. Although little research has specifically addressed acceptability for adolescents, some research shows that that psychotherapy may be more palatable than medication [9]. Considering that health behaviors developed during adolescence can establish a trajectory for future health outcomes, [20, 21] providing effective and acceptable interventions for this population could have long-term benefits. Adolescents who do not receive adequate mental health treatment are more likely to experience poor health, low social support, work impairment, decreased marital satisfaction, and reoccurring depression in adulthood [20–22].

In recent decades, innovative treatments have focused on addressing depression through a more holistic approach (e.g., increased attention to interactions of physical, mental, spiritual, and social health), [23–25] rather than using traditional therapies that more narrowly focus on symptom reduction (e.g., Cognitive Behavioral Therapy, Interpersonal Therapy) [26]. Mind-body practices constitute a form of integrative medicine that has grown in popularity [27]. Defined as a diverse collection of complementary practices (e.g., guided imagery, biofeedback, meditation), [28] mind-body medicine has been linked to improvements in chronic pain, anxiety and depression [29, 30]. The Center for Mind-Body Medicine (CMBM) has developed a comprehensive treatment model called Mind-Body Skills Groups (MBSGs) that incorporates a variety of mind-body practices into one integrated package [31]. The mission of the CMBM program is to maximize self-awareness and emotion-regulation through the use of evidence-based self-care tools and techniques. The MBSG treatment has shown to be successful in reducing symptoms of posttraumatic stress disorder [32] and depression for youth [33, 34], including treating depression in a primary care settings [34]. However, less is known about its perceived acceptability.

Given that acceptability is a key predictor in determining success in future implementation and improving outcomes, [3–5] understanding whether programs like MBSGs are appealing to depressed adolescents is crucial. The objective of this study was to investigate adolescents’ perspectives on the acceptability of MBSGs as a depression treatment in primary care.

## Methods

### Recruitment and procedures

This project used data from a pilot study examining the preliminary effectiveness for MBSGs delivered to depressed adolescents in primary care [34]. Adolescents were identified by behavioral health clinicians who were embedded in three participating primary care clinics in an urban county hospital system. This public hospital system serves a large metropolitan area, with 10 primary care locations and sliding scale fees to allow easy access to care. Adolescents who were identified as having depression during a primary care visit **-** and who expressed interest in the study **-** met with a research assistant to discuss the study procedures and to assess eligibility. Inclusion criteria required that adolescents (1) screened positive for depression based on a structured diagnostic interview assessment, [35] (2) were primary care patients of the participating hospital system, (3) were between 13 and 17 years old, (4) spoke English, and (5) were willing to attend the MBSG intervention for 10 consecutive weeks. Adolescents were excluded if they (1) had a history of bipolar or psychosis, (2) were at an acute and immediate risk for suicide at the time of screening, (3) and/or had previously participated in a MBSG.

MBSGs took place at participating primary care clinics and groups typically consisted of eight to ten adolescents. The MBSGs were held once per week for 10 weeks; each session lasted approximately 1.5 h after school and followed a structured outline [36]. Each session was led by two female masters-level social workers who were trained to deliver the MBSG intervention and who also served as behavioral health clinicians at the participating primary care clinics. Behavioral health clinicians met once a week to review the MBSG training manual with a certified CMBM supervisor. During the 10-week series, each group was taught a variety of skills/activities to help foster self-awareness and emotion regulation, including: guided imagery, autogenic training, biofeedback, meditation, genograms, mindful eating, and self-expression through drawing and words [34]. Adolescents completed a short semi-structured interview to measure acceptability at the end of the MBSGs (post-intervention) and again 3 months later (follow-up). Interviews took place at the primary care clinic in which they received the treatment. Adolescents were compensated for each study-related visit they attended (baseline, post-intervention, and follow-up) and for attending each MBSG sessions. Indiana University’s Institutional Review Board and the participating hospital system’s research committee approved the project. The CMBM trial was registered at the United States National Institutes of Health (Clinical Trials.gov; NCT03363750) and adhered to clinical trial guidelines.

### Measures

Adolescents were asked to report their age, race, gender, ethnicity, and grade level at baseline. A short battery of outcome measures were included [34]. In addition, at post-intervention and 3-month follow-up, participants were asked 4 open-ended interview questions regarding their experience in the MBSGs. These questions were developed by the research team and are the focus of the current analysis. Interview questions can be found in Supplemental Table 1 and include the following: *(1) Did the Mind-Body Skills Group help you? If so, how? (2) Was there anything about the Mind-Body Skills Group you didn’t like? If so, what? (3) What was the most important and interesting part of the group? Why was it important and interesting? and (4) Has your life or your outlook on the world changed because of the group? If so, how?* Responses were recorded in writing by research staff.

### Qualitative analysis

Qualitative data was analyzed using an inductive, consensus-based approach that borrowed concepts from grounded theory [37] and other qualitative techniques [38, 39]. Analysis began by research staff (LJ, ES & MS) individually reviewing a small subset of responses and developing independent coding schemes. The team met to discuss their findings and to agree upon emerging themes in which to categorize responses. Iterative, consensus-building conversations continued until data collection stopped and a final codebook was developed. Using the final codebook, research staff (LJ & ES) divided the interviews in half and individually coded their assignments. Once independent coding assignments were complete, the research staff (LJ & ES) reviewed each other’s work to check for consistency and accuracy. Any discrepancies found were flagged and discussed until mutual agreements on the coding were met. Below we present descriptive data and highlight differences between the two follow-up periods.

## Results

**Fig. 1:**
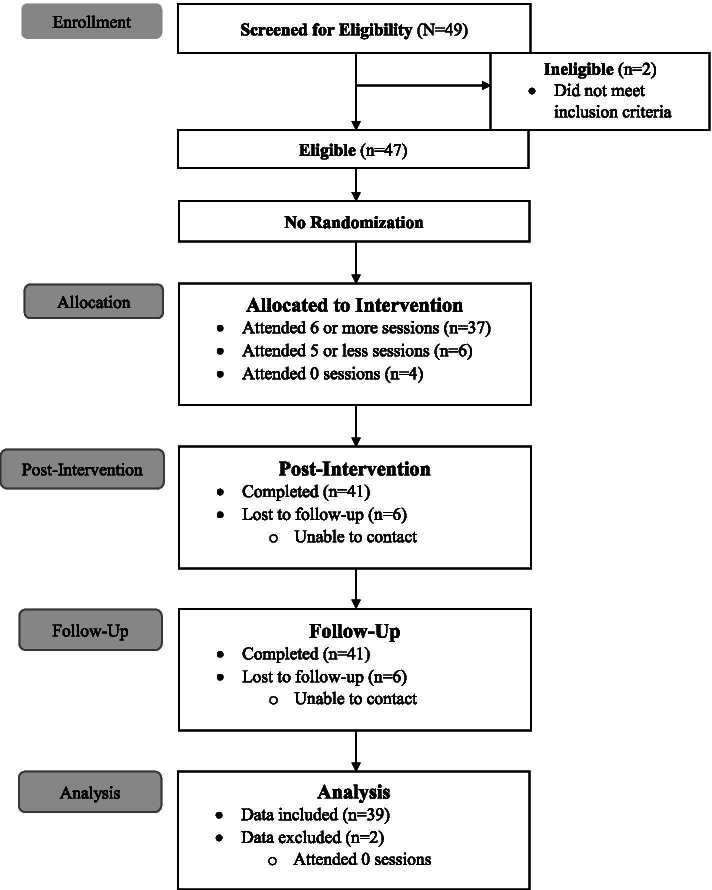
Flow Diagram

A total of 49 adolescents were recruited to participate in the study; 47 enrolled, and 41 completed the semi-structured interview at the post-intervention and 3-month follow-up visit. Of the 41 participants with complete follow-up data, 2 did not attended any group sessions and were therefore excluded from the current study (Fig. 1). The majority of participants were female (76%), Hispanic/Latinx (69%), and in Junior High (61%; Table 1).

**Table 1:** Adolescent characteristics

Characteristics	Frequency	Percent
**Age** (M = 15.1; SD = 1.1)		
**Gender**		
Female	30	76.9%
Male	9	23.1%
**Race**		
White or Caucasian	16	41.0%
Black or African American	6	15.4%
Multiracial	2	5.1%
Refused or Unknown	15	38.5%
**Ethnicity**		
Hispanic or Latinx	27	69.2%
Not Hispanic or Latino	11	28.2%
Unsure	1	2.6%
**Education Level**		
Junior High (8th - 9th grade)	24	61.5%
High School (10th – 12th grade)	15	38.5%

At both the post-intervention and 3-month follow-up, 84% of adolescents reported that the MBSGs were helpful to them. Few adolescents reported that the groups did not help them or were unsure whether the groups helped. When asked at post-intervention how the MBSGs helped them, nearly half reported that the skills/activities they learned during the group aided them (see Table 2); fewer adolescents reported this at the follow-up. Additionally, adolescents reported that social relationships built during the group were also helpful. At both time points, a consistent number of adolescents described positive outcomes they experienced because of the group (38%), including enhanced coping strategies and improved insight into what was causing their depression.

**Table 2:** Adolescents’ Responses to Semi-Structured Interview *N* = 39

Key Concept	Post-Intervention n (%)	Follow-up n (%)	Representative Quote
***How did the Mind-Body Skills Group help you?***			
Skills or Activities	19 (49)	15 (38)	“All the skills they taught us really did help once we applied them. They did help us all cope with certain things.”
Outcomes	15 (38)	15 (38)	“...it helped me relax and figure out why I was always upset … It helps me relax and stay in control.”
Socializing and Connection to Others	16 (41)	11 (28)	“I feel very connected with the people in the group. This connection with people helped me because I know I have someone to count on.”
Other	4 (10)	6 (15)	“They taught us skills, but they didn’t help as much with me. Maybe for others, but not me.”
***What was the most important and interesting part of the group?***			
Skills or Activities	28 (72)	17 (44)	“Going each week and learning a new skill was interesting because it taught me how to handle being overwhelmed with emotions.”
* Drawing*	*9 (23)*	*4 (10)*	*“The drawings on week 1 and week 10. It revealed a lot about each and every kid in there.”*
* Meditation*	*4 (10)*	*7 (18)*	*“The meditation. It helped me focus more on things and made me feel calm.”*
* Soft Belly Breathing*	*4 (10)*	*0 (0)*	*“It was also interesting to see how the breathing helped everyone.”*
Socializing and Connection to Others	22 (56)	24 (62)	“Hearing other people’s stories. The fact that other people go through the same type of stuff. It makes me feel like I don’t want anything to happen to them, and they feel the same towards me.”
Other	5 (8)	4 (10)	“Learning how to have a mind and body connection was the most interesting.”
***How has your life or outlook of the world changed?***			
More Positive Thinking/Less Negative Thoughts	15 (39)	17 (44)*	“I have become more hopeful and I feel like I have better chances for my future.”
Socializing and Connection with Others	15 (39)	16 (41)*	“Before I felt that people didn’t like me. But now I have learned how to communicate and relate with others. Feels like I have more friends now”.
Increased Self-Worth	6 (16)	3 (8)*	“The groups helped me realize I matter more than I thought”.
Increased Emotional Regulation	6 (16)	5 (13)*	“I’m more calm and can control my emotional distress. I don’t feel as lost as I did before.”
Unsure if Helped	4 (11)	2 (5)*	It really allowed me to understand things in my life from a different perspective but didn’t really change how I felt about them.”
Did Not Help	3 (8)	5 (13)*	“I feel stuck in the middle of things. I can see two sides of life, but I don’t know which side to choose.”
Reduced Suicidal Ideation	3 (8)	2 (5)*	“I don’t feel like killing myself at all…”
Other	3 (8)	6 (15)*	“I don’t like expressing my feelings.”

Note: Categories are not mutually exclusive. Percentages can be more than 100%

*N = 38 participants. One individual chose not to answer this question

When asked if there was anything they did not like about the group, close to half of the adolescents said no; this percent increased at the 3-month follow-up visit. When adolescents disliked an aspect, they most frequently mentioned certain skills/activities, including active meditation, quiescent meditation, and genograms skills. A few reported not liking the length of the group, primarily because they wanted the group to last longer than 10 weeks and others stated that they did not like the time of the group (groups started anywhere between 3 and 4 PM), primarily because it interfered with academic and extracurricular activities.

Two themes emerged when adolescents were asked what the most important and interesting part of the MBSGs was: the group skills/activities and the social connection between group members. At post-intervention, the majority of adolescents described the skills/activities as important and interesting (72%), with drawing being the most popular response (Table 2). However, at the 3-month follow-up, more adolescents found the social connection between other group members to be the most important and interesting part of the group - compared to the skills/activities, which showed a decrease over time (Table 2). Adolescents who talked about the skills/activities said the activities were enjoyable, provided emotion regulation and personal insight, and presented new ideas that they had never learned before attending the group. Adolescents who talked about the social connection being important reported that the groups provided opportunities to build new friendships and express one’s true feelings in an environment that was comfortable, supportive, and that elicited solidarity.

Adolescents reported that the MBSGs changed their life or outlook on the world following the intervention (72%); however, this percent decreased slightly at the follow-up visit (61%). When asked how their life or outlook had changed, the two most common responses involved increased positivity (fewer negative thoughts and increased hopefulness) and social connection with others (feeling less isolated from others and being able to connect or trust others more easily). Additionally, many described experiencing increased self-worth), emotion regulation and decreased suicidal ideation. Few adolescents were unsure or did not think that the MBSGs changed their outlook on life.

## Discussion

Acceptability is an important element to consider when assessing the implementation of a new treatment; without establishing strong acceptability, new interventions can be costly to patients, providers and institutions, [40] and result in low adherence and participation [41]. Adolescent mental health treatment adherence is often challenging, [18, 42, 43] which makes it even more important to identify engaging interventions. Results from our study suggest that MBSGs may be an acceptable treatment for adolescents coping with depression. In light of positive improvements in depression, [34] adolescents in our study also reported positive perceptions of the MBSGs. These groups provided useful strategies to help combat depressive symptoms and also generated a sense of belonging and connectedness with others. After the intervention, adolescents reported several positive outcomes related to the group. For example, many adolescents reported feeling more positive, hopeful, and valued as an individual, while also feeling less isolated, depressed, and suicidal. Positive emotions, such as hope and connectedness have shown to be inversely related to depression, [44–46] and also predictive of life satisfaction, high self-esteem and optimism [47, 48]. Results from our study reinforce the significance of eliciting trust, hope, and social connection when establishing acceptability of behavioral health treatments for youth.

Our results also found that a small number of adolescents reported disliking the groups, primarily because of specific activities practiced in the group. Yet others identified some of the same activities as beneficial. This reinforces the idea that offering different techniques – rather than focusing on one modality – can be beneficial. Offering a diverse array of practices in one comprehensive package ensures that every individual can explore and determine which techniques are most useful and appropriate for their personal needs. A packaged approach, with several types of mind-body practices may be particularly beneficial for youth, given that adolescence is a period characterized by autonomy seeking behaviors and exploration of different identities, values and secular interests [14, 49].

The nature of adolescent development and the heightened sensitivity for social influence during this time is complex. Therefore, it is imperative to provide treatments that complement the developmental challenges experienced during this transitional period [50]. MBSGs help facilitate developmental needs by providing adolescents the autonomy to choose which self-care techniques they prefer, and ultimately allow them to learn more about themselves and their treatment preferences. Additionally, a strength of the MBSGs is its ability to elicit a therapeutic partnership between group members and facilitators. Group facilitators provide didactic instruction to educate and support adolescents; yet, also refrain from threating an adolescents’ authority. Support for this can be found in our study results, in which several adolescents reported that the MBSGs were important and interesting because it provided a safe space to develop individualistic ideas and friendships. Similar findings were also reported in prior research studies exploring the effects of MBSGs on youth coping with depression, anxiety, and post-traumatic stress disorder [32, 33]. Therefore, the blend of didactic and interactive instruction, along with the strong emphasis on treatment preference autonomy, may help explain why adolescents found this treatment acceptable. Moreover, the program’s ability to foster social support and positive relationships appeared to be a reoccurring and important theme during interviews. Cultivating strong social support in healthcare settings has been linked to increased treatment adherence, [51] and research finds that social support can also positively impact health outcomes related to depression [52, 53]. Thus, another plausible explanation to the treatment’s acceptability could be related to the social connections with others in the MBSGs - which may have also helped improve symptoms related to their depression [34].

Although the majority of adolescents in our study had positive things to say about the program, some participants did suggest areas of improvement for future development. Feedback frequently included discussions about the timing of the groups, when the groups occurred, and how long the groups lasted. Some adolescents reported concerns about the groups taking place after school, mainly because of transportation and interference with after school activities, which are common barriers to adolescent treatment adherence [54]. Efforts should be made to help accommodate family needs by ensuring programs are highly accessible in a variety of locations, times, and formats. It was also suggested that the length of the groups be extended, as a few participants disclosed that they felt rushed for time. Feedback for ways to improve this area included having groups meet longer than 10 weeks, meet longer than 1.5 h, or meet twice a week. This finding is encouraging given that adolescents may be challenging to engage in treatments, [55] and suggests that at least for some participants, extended time would be helpful for learning the mind-body practices.

Lastly, given the large proportion of Hispanic/Latinx youth in our sample, MBSGs may be particularly promising for engaging the Hispanic/Latinx community. Hispanic/Latinx youth are more likely to experience depression compared to other races or ethnicities, [56, 57] yet are less likely to engage in mental health treatments [8]. Research on culturally sensitive, tailored interventions for this population is still in its infancy, although preliminary evidence has shown the clear benefit of interventions that resonate with core values held by Hispanic/Latinx groups [58, 59]. For example, group-based therapy like MBSGs may resonate with Hispanic/Latinx populations due to this culture’s embrace of collectivist ideologies and togetherness, called *familismo* [60, 61]*.* Considering that participants in our study were largely Hispanic/Latinx and female - and that engagement and acceptability within our study was strong - our results may provide preliminary evidence that MBSGs are acceptable for this particular population.

### Limitations

This pilot study begins to establish acceptability of MBSGs as an intervention in primary care for adolescents with depression. However, results should be interpreted with caution. Given the relatively small and uniform sample - primarily female (79%) and Hispanic/Latinx (67%) - who were recruited from three clinics in one centralized hospital system, our findings may not generalize to all adolescents seeking mental health treatment in primary care. Larger scale studies, with more diverse samples and settings are needed. Future studies should also consider acceptability of MBSGs from other stakeholders, such as clinicians who deliver the treatment or from parents/guardians of adolescents receiving the treatment. Adolescents are embedded within a larger system, and other stakeholder views will be important for future implementation efforts. Finally, our investigation was limited by measurement concerns. Data came from four open-ended questions that covered basic domains of acceptability and did not include more in-depth questioning about their experience. Although interviewers were independent of treatment providers, participants may have wanted to please the interviewers given these were the same people who recruited and initially interviewed them.

## Conclusion

Establishing acceptability is a crucial step in developing targeted interventions to prevent negative health outcomes for adolescents with depression. Mind-body modalities, such as MBSGs, have recently gained interest as an innovative treatment option for this population, given that the large amount of flexibility built into these interventions may resonate with important adolescent values such as autonomy and connectedness. Results of this study indicate that most adolescents found the MBSGs to be helpful and facilitated positive changes in their outlook on the world. Importantly, those who cited unhelpful aspects of the intervention tended to cite specific techniques and skills as opposed to overall concerns with the intervention, which is in line with the customizable, integrated approach of MBSGs. Future randomized trials will be important to assess comparative acceptability of this intervention to usual standards of care in primary care settings and more fully characterize its integration into these settings.

## Supplementary Information

**Additional file 1: Supplemental Table 1**. Semi-Structured Interview Questions.

## Data Availability

The datasets used and/or analyzed during the current study are available from the corresponding author on reasonable request.

## Abbreviations

CMBM: Center for Mind-Body Medicine
MBSGs: Mind-Body Skills Groups
