# Supplementary material for: Do adolescents consider mind-body skills groups an acceptable treatment for depression: results from a pilot study
Source: BMC Pediatr. 2021 Oct 27;21:475. doi: 10.1186/s12887-021-02942-3 (PMC8549145; doi:10.1186/s12887-021-02942-3)
Supplement: Supplementary file 1 — Additional file 1: Supplemental Table 1. Semi-Structured Interview Questions. [file 12887_2021_2942_MOESM1_ESM.docx]

**Supplemental Table 1.** Semi-Structured Interview Questions

| 1. Did the Mind-Body Skills Group help you?    1. If so, how? |
| --- |
| 1. Was there anything about the Mind-Body Skills group you didn’t like?    1. If so, what? |
| 1. What was the most important and interesting part of the group?    1. Why was it important and interesting? |
| 1. Has your life or outlook on the world changed because of the group?    1. If so, how? |
